# Polysaccharides as Green Fuels for the Synthesis of MgO: Characterization and Evaluation of Antimicrobial Activities

**DOI:** 10.3390/molecules28010142

**Published:** 2022-12-24

**Authors:** Nayara Balaba, Silvia Jaerger, Dienifer F. L. Horsth, Julia de O. Primo, Jamille de S. Correa, Carla Bittencourt, Cristina M. Zanette, Fauze J. Anaissi

**Affiliations:** 1 Departamento de Química, Universidade Estadual do Centro-Oeste, Guarapuava 85040-080, Brazil; 2 Chimie des Interactions Plasma-Surface (ChIPS), Research Institute for Materials Science and Engineering, University of Mons, 7000 Mons, Belgium; 3 Departamento de Engenharia de Alimentos, Universidade Estadual do Centro-Oeste, Guarapuava 85040-080, Brazil

**Keywords:** eco-friendly synthesis, *Aloe vera*, starch, MIC, bacteria, antifungal

## Abstract

The synthesis of structured MgO is reported using feedstock starch (route I), citrus pectin (route II), and *Aloe vera* (route III) leaf, which are suitable for use as green fuels due to their abundance, low cost, and non-toxicity. The oxides formed showed high porosity and were evaluated as antimicrobial agents. The samples were characterized by energy-dispersive X-ray fluorescence (EDXRF), X-ray diffraction (XRD), Fourier-transform infrared spectroscopy (FTIR), and scanning electron microscopy (SEM). The crystalline periclase monophase of the MgO was identified for all samples. The SEM analyses show that the sample morphology depends on the organic fuel used during the synthesis. The antibacterial activity of the MgO-St (starch), MgO-CP (citrus pectin), and MgO-Av (*Aloe vera)* oxides was evaluated against pathogens *Staphylococcus aureus* (ATCC 6538P) and *Escherichia coli* (ATCC 8739). Antifungal activity was also studied against *Candida albicans* (ATCC 64548). The studies were carried out using the qualitative agar disk diffusion method and quantitative minimum inhibitory concentration (MIC) tests. The MIC of each sample showed the same inhibitory concentration of 400 µg. mL^−1^ for the studied microorganisms. The formation of inhibition zones and the MIC values in the antimicrobial analysis indicate the effective antimicrobial activity of the samples against the test microorganisms.

## 1. Introduction

Magnesium oxide (MgO) is an ionic material with a refractory capacity to withstand high temperatures [1]. It presents a well-defined crystalline structure of face-centered cubic, and, when hydrated, it converts to its hydroxide form (Mg(OH)_2_) [2]. MgO is a functional, low-cost, environmentally safe metal oxide applied in various industrial fields, e.g., the plastics, rubber, paper, and adhesive industries; in agriculture, MgO is incorporated into animal feed and fertilizer, and it is used in refractory applications in steel production and equipment coatings [3]. MgO has also been studied for the adsorption of textile dyes, metal ions, and phosphates in the catalysis applied in ceramic materials and paints [1,2,3,4,5,6,7]. Several methodologies for the synthesis of MgO can be found: precipitation [2], microwave-assisted [4,8], sol–gel [9], and hydrothermal [5]. The main production method is by calcining dolomite (CaMg(CO_3_)_2_) and brucite (Mg(OH)_2_); through this process, decomposition occurs at a high temperature [3]. However, the MgO obtained with this process is divergent due to the low specific surface area, irregular morphology, and grain size [3]. Reports on other methodologies for the synthesis of structured magnesium oxide are available [2,4,5] but the search for organic fuels to be used in synthesis has been considered of importance due to their ecological origin, easy access to natural polysaccharides, low cost, high chemical reactivity, high combustion power, reduction in the calcination temperature, and action as a complexing gelling agent [10].

In the present work, starch, citrus pectin, and *Aloe vera* leaf were used as fuels to obtain structured MgO by the combustion method. In synthesis route I, starch extracted from cassava (*Manihot esculenta*), a high-energy tuber and a low-cost, biodegradable polysaccharide from renewable raw material sources, consisting of amylose and amylopectin molecules that are composed of D-glucose units, was used [11]. In route II, citrus pectin, a polysaccharide derived from the peels of citrus fruits (lemon, orange, etc.) used in the food and pharmaceutical industries, due to its high gelling capacity, was used [12]. It consists of α-D-galacturonic acid units joined by glycosidic bonds (α-1,4) and esterified methyl carboxyl groups [12]. Finally, *Aloe vera* (*Aloe Barbadensis Miller*) was used in route III, a succulent perennial of the family Liliaceae, used for its pharmacological properties [13]. According to Hamman (2008), the *Aloe vera* leaves have three structural components: the cell walls, the degenerated organelles, and the viscous liquid contained within the cells. These components present many compounds, such as proteins, lipids, amino acids, vitamins, enzymes, inorganic compounds, small organic compounds, and polysaccharides. Among the polysaccharides, one can find mainly mannose, cellulose, and pectic polysaccharides, whereas the skin of the leaf contains, in addition, significant quantities of xylose-containing polysaccharides [13,14]. Describing a reaction mechanism for the synthesis of materials with *Aloe vera* is complex due to the different compounds present in the plant extract [15]. However, a sustainable reaction mechanism for the formation of material with organic fuel is the interaction of metal ions that bind with biomolecules through functional groups and π electrons by ionic bonds or van der Waals forces; this depends on the concentration of plant extracts [15]. The synthesized MgO samples were characterized, and their biological activities were studied.

Antimicrobial resistance (AMR) is a global threat to human health, causing thousands of deaths annually [16]. One of the leading causes of drug-resistant pathogens is the excessive exposure of microorganisms to antibiotics as a treatment against infection [17,18]. Another problem caused by microorganisms is biofilms, which are aggregates of cells embedded in a self-producing matrix of extracellular polymeric substances (EPS), which adhere to each other and/or to a surface and have greater potential to survive in adverse conditions and end up generating resistance to antibiotic treatment and the host’s immune system [19]. Bacteria and diverse fungi show greater susceptibility to antibiotic resistance due to some strains’ evolutionary and adaptive conditions [17,20]. For example, the bacterium *Staphylococcus aureus*, responsible for infections such as postoperative wounds and prosthetic infections related to endotracheal tubes and other biomaterials, has been reported to cause nosocomial infections and AMR to several drugs, such as penicillin, methicillin, quinolone, and vancomycin [17,21]. A fungus that has become resistant to antibiotics is *Candida albicans*, an opportunistic pathogen, generally harmless to human beings, which can be found on the surface of humid mucous, such as in the intestine, vagina, and oral cavity [17]. The fungus *C. albicans* shows drug resistance to amphotericin B and azoles [22], two known antifungal agents. However, colonization, infectious aggravation, and damage to the structure of cells may occur with the weakening of the host’s immune system [17]. Therefore, it is necessary to develop alternative strategies to minimize the problem of AMR, such as materials that inhibit the growth of microorganisms, preventing contagion with infectious diseases [23]. In this context, some papers present antimicrobial studies, as Al-Shammar et al. (2021), which uses zein nanoparticles loaded with transition metal ions to control three *Candida* species [24], and metal oxide particles have presented a wide range of biological applications, such as drug and gene delivery and cell, tissue, and diagnostics engineering, as well as limiting the growth of microorganisms [4]. Reports on structured magnesium oxide (MgO) describe its efficiency in inhibiting the growth of food and aquatic pathogen colonies and controlling the proliferation of bacteria and fungi, attracting significant interest due to its non-toxic nature [4,5,25]. The common mechanism of the antibacterial activity of MgO is due to the oxygen vacancies, leading to the higher production of reactive oxygen species (ROS) (OH^−^, O^2−^, and H_2_O_2_) on the surfaces of the particles when oxidative stress occurs on the bacterial cell wall, leading to cell death [4]. Another possible mechanism is when Mg^2+^ ions are released from MgO with irregular morphology and rough edges, coming into contact with the cell membrane of the microbe [4,8]. The negatively charged cell membrane and Mg^2+^ attract each other and the Mg penetrates the cell membrane and damages it, leading to cell death [8]. Therefore, in this study, three natural polysaccharides were used as precursors to the combustion reaction to obtain structured MgO, aiming at its application as an antibacterial and antifungal agent.

## 2. Results 

### 2.1. Characterization of Synthesized MgO Particles

According to the semiquantitative analysis (EDXRF) (Table 1), the sample with the highest elemental magnesium percentage was MgO-St, with 86.6% atomic, while MgO-Av presented 64.1%, and MgO-CP 76.2% (Figure S1). This difference can be associated with the composition of the organic additives: starch contained ~6.6% atomic Mg ions, citrus pectin presented ~1.7% of magnesium, and *Aloe vera* gel contained ~0.1% (Figure S2). The composition of the organic additives varied according to the region from which they were harvested [26]. The samples’ composition variation indicates that the organic fuel used in the synthesis influences the amount of metal ions in the material obtained, interfering with the physicochemical properties and affecting the performance against bacteria and fungi.

**Figure 1:**
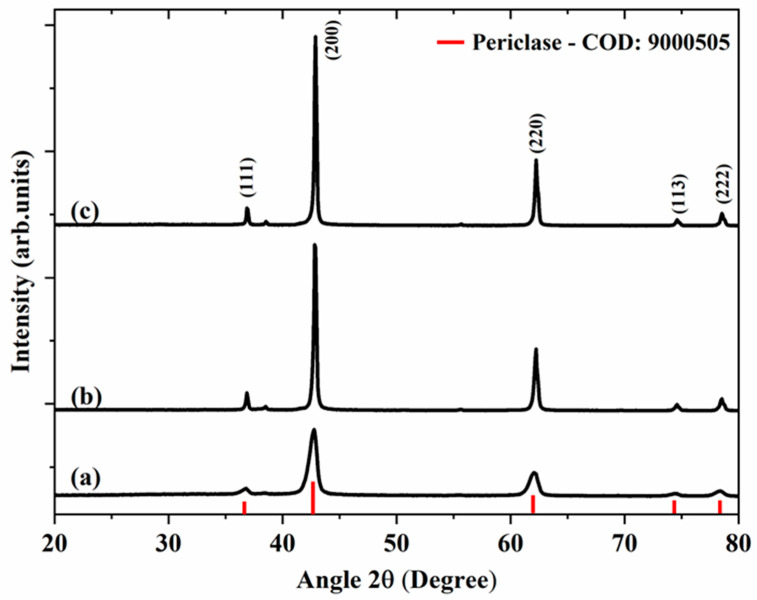
XRD patterns of samples MgO-St (**a**), MgO-CP (**b**), and MgO-Av (**c**).

Figure 1 shows the X-ray diffractograms of magnesium oxide samples obtained from starch (Figure 1a), citrus pectin (Figure 1b), and *Aloe Vera* (Figure 1c). In all samples, peaks (111), (200), (220) (113), and (222) of the periclase crystalline phase, the typical phase of the face-centered cubic MgO (COD: 9000505), were observed [27,28].

A few differences between the diffractograms were observed when using different fuels. The samples showed different peak intensities, mainly peak (200) at 42.6° and peak (220) at 61.8°, and MgO-St was found to have a greater width at the average height compared to the peaks of the MgO-CP and MgO-Av diffractograms, which would suggest a smaller crystallite size [28].

**Figure 2:**
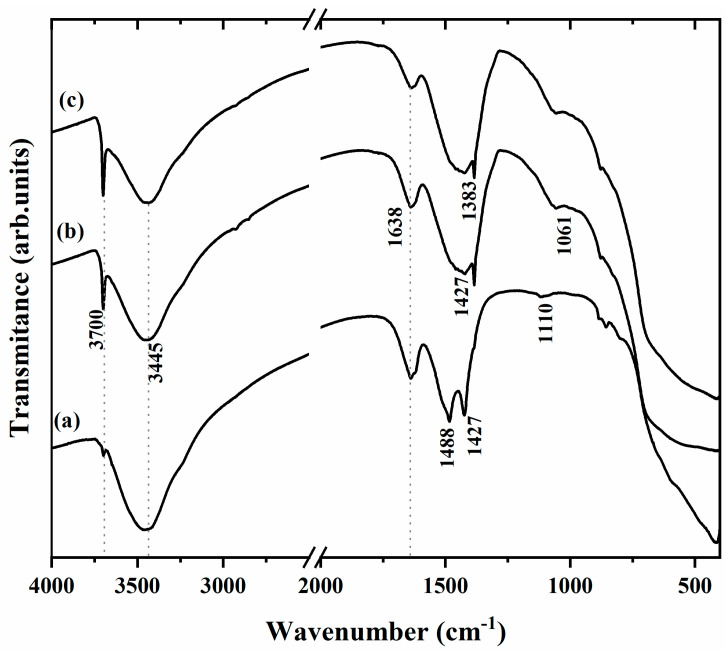
FTIR spectra for the samples (**a**) MgO-St, (**b**) MgO-CP, and (**c**) MgO-Av.

The FT-IR spectra of the MgO samples are shown in Figure 2. The IR spectra exhibit a narrow and intense band at 3700 and 3445 cm^−1^, attributed to the vibrational stretching of the free -OH ions of Mg(OH)_2_, generated by the hydration of MgO [29]. The γ(OH) region shows two bands at 1488 cm^−1^ for the MgO-St sample (Figure 2a) and 1638 cm^−1^, which correspond to the O–H bending mode, which is characterized by the bending vibration of the -OH group of the physiosorbed water molecules [29,30]. The broad bands at 1427 and 1383 cm^−1^ for MgO-St, MgO-CP, and MgO-Av (Figure 2b,c) are assigned to the asymmetrical and symmetrical stretching vibrations of CO_2_ species chemisorbed onto the surface of MgO [31,32]. The low-intensity bands at 1110 and 1061 cm^−1^ observed for MgO-CP and Mg-Av can be associated with the presence of H ion species as defects of octahedral symmetry, characteristic of magnesium oxides calcined between 700 and 800 °C [33,34].

**Figure 3:**
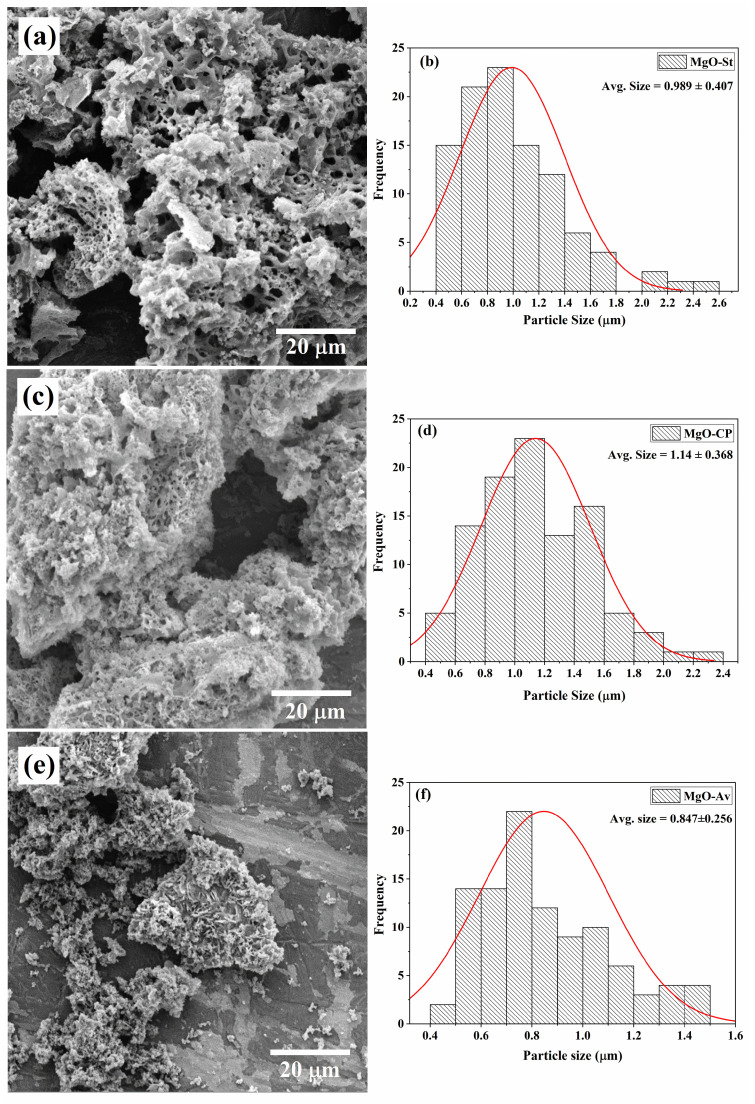
SEM images of synthesized MgO using different natural additives as fuel sources and average particle sizes of the samples: (**a**,**b**) MgO-St, (**c**,**d**) MgO-CP, and (**e**,**f**) MgO-Av.

Scanning electron microscopy was used to study the morphology of the synthesized samples (Figure 3). The morphology of the MgO-St sample (Figure 3a) displayed a structure composed of non-uniform pores and hole voids, with spongy characteristics in the material, with an average particle size of approximately 0.99 µm (Figure 3b). The holes may be due to the large number of gases released during the combustion of the reagents and the starch used in the synthesis. These voids and pores provide a large surface area, supporting antimicrobial activity [35]. Figure 3c shows SEM images of the MgO-CP sample, which had an irregular morphology, forming small pseudo-spheres and hole voids [20], showing larger particles than the MgO-St sample, with an average size of 1.14 µm (Figure 3d). Finally, the morphology of the MgO-Av sample (Figure 3e) was irregular and composed of periodic sheets, and the sample consisted of small particles with an average size of 0.85 µm (Figure 3f). The different morphologies observed in the samples are attributed to the different polysaccharides used in each type of synthesis, which will affect the antimicrobial properties of each material [35,36,37].

### 2.2. Evaluation of Antimicrobial Activity

**Figure 4:**
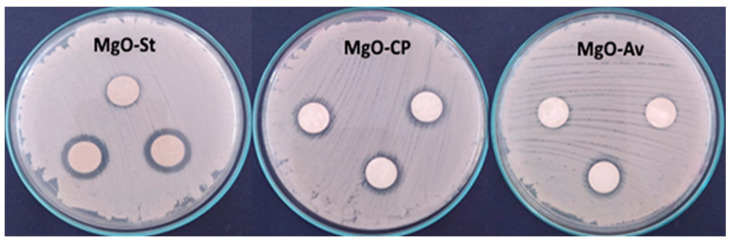
Photographs of halos of inhibition formed in tests against *C. Albicans* using the oxide samples MgO-St, MgO-CP, and MgO-Av. The oxide MgO-St showed the largest halo of inhibition.

The MgO samples were tested against *C. albicans* (ATCC 64548) using the disk diffusion test; see Figure 4. The clear zones around the specimens indicate the inhibition of the fungal growth. This halo of inhibition (in millimeters) was used to quantify the antifungal activity obtained, and their averages are presented in Table 2. The sample with the highest antifungal activity is the one with the largest halo around the disk; the diffusion of the antimicrobial agent leads to the formation of a zone of inhibition of bacterial growth, whose diameter is proportional to the inhibition [38]. The MgO-Av sample shows weak antifungal activity. In contrast, the MgO-St sample exhibits an inhibition zone with a diameter of 3.0 mm on average. The MgO-St samples have the largest surface area. Several studies have correlated a large surface area with good antibacterial and antifungal activity performance [20], suggesting that the superior inhibition observed for the MgO-St sample is associated with its surface characteristics and morphology.

**Figure 5:**
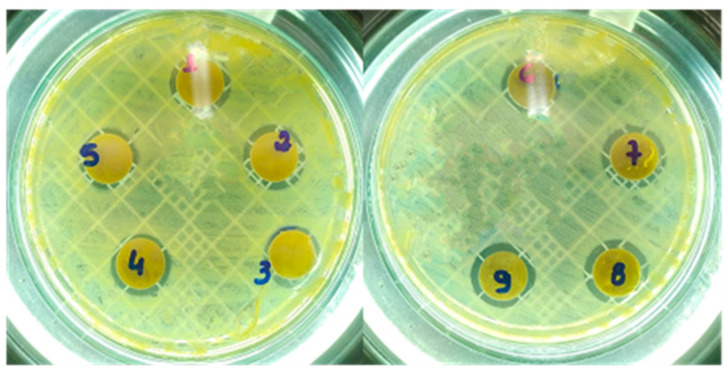
Photographs of halos of inhibition formed in tests against *Staphylococcus aureus*. *MgO-CP (labeled as 1,2,3); MgO-Av (labelled as 4,5,6); MgO-St (labeled as 7,8,9).

MgO is reported to have antibacterial properties, including excellent antibacterial activity against *K. pneumonia* bacteria [35,39]. In this context, we investigated its antibacterial activity against *S. aureus* and *E. coli*; the MgO-synthesized samples exhibited higher inhibition than the control (Figure 5).

The MgO samples were tested against pathogenic Gram-positive *S. aureus* and Gram-positive *E. coli* bacteria using the disk diffusion methodology. Table 2 shows the means and standard deviations of the inhibition zone; the halo or zone of inhibition can be observed in Figure 5 by the darker zones around the disks. It was found that all the MgO samples had antibacterial properties against *S. aureus*. The MgO-St sample had statistically higher activity against *S. aureus* and *C. albicans*, with a larger inhibition diameter, which might explain its larger surface area compared to the other samples (MgO-Av and MgO-CP).

It has been reported that the antibacterial activity increases with the decreasing size of nanoparticles due to the bacteria MgO surface interaction, which depends on the surface area available [20]. Against *S. aureus*, the oxides synthesized with citric pectin and *Aloe vera* fuels were statistically equal; however, against the *C. albicans* fungi, the oxide MgO-CP showed significantly higher activity than the MgO-Av sample. The MgO samples were ineffective against *E. coli*, which can be associated with the Gram-negative bacterial composition of a thin peptidoglycan cell wall and an outer membrane containing lipopolysaccharide, leading to higher resistance than for Gram-positive bacteria such as *S. aureus* [20]. Some studies, such as Umaralikhan and Jaffar, also used *Aloe vera* leaves as a fuel source for MgO synthesis and tested them against *S. aureus* and *E. coli* as an antibacterial material in the disk diffusion method; they obtained considerable inhibition success against both bacteria [25]. The diffusion of agar disks is a qualitative assay used to test bioactivity in a sample. However, in this method, the effect of antimicrobial activity is not accurately estimated [40].Therefore, the synthesized oxides were tested by the minimum inhibitory concentration (MIC) method to quantify the inhibitory concentration for each strain. The values for the MIC provide a quantitative evaluation of the antimicrobial action of the samples against *E. coli* and *S. aureus* pathogens. The MIC is defined as the lowest concentration (µg.mL^−1^) of an antimicrobial agent, which, under strictly in vitro conditions, completely prevents the growth of the test strain of an organism (EUCAST, 1998) [41]. The MIC of each sample showed the same inhibitory concentration of 400 µg. mL^−1^ for both bacteria *S. aureus* and *E. coli,* and also showed a minimum inhibitory concentration for *C. albicans* of 400 µg.mL^−1^. The broth microdilution method allowed the quantification of the minimum inhibitory concentration; however, the same did not occur in the agar diffusion disk tests. It can also be observed that, unlike the broth microdilution method, the three samples did not present inhibitory activity in the disc diffusion tests against *E. coli* bacteria. The absence of the inhibition zone does not necessarily indicate that the sample is inactive against the tested strain but that the diffusion may be incomplete; this occurs especially in compounds that diffuse more slowly in a solid culture medium or due to the lipophilic characteristics or/and the chemical nature of the isolated substances [42,43].

The antibacterial efficacy of MgO samples has been explained by a set of factors that inhibit bacterial growth, such as the production of reactive oxygen species (ROS) due to the presence of Mg^2+^, the interaction between MgO particles and the membrane cell wall, and the penetration of individual particles into cells [39]. The antibacterial properties may also be associated with the correlation between the surface area and volume of oxide particles, which form more active oxygen species outside the bacterial cell, destroying the bacteria’s cell membranes [8,20].

## 3. Discussion

The samples synthesized in this work showed differences in their structural, morphological, and antimicrobial properties, associated with the different polysaccharide sources used in each type of synthesis. The three MgO samples presented the same crystalline structure of the face-centered cubic type, characteristic of magnesium oxides; however, the MgO-St had a smaller crystallite size compared to MgO-CP and MgO-Av. The same structure was reported by Umaralikhan and Jaffar [25] and El-Shaer et al. [44]. El-Shaer et al. synthesized MgO using the conventional sol–gel method and annealed it at different temperatures for 5 h; the samples calcined above 500 °C showed similar antimicrobial activity results as in this work. According to these authors, the calcination temperature affects the number of surface defects and therefore the antimicrobial properties [44], justifying the antimicrobial results presented for the MgO-St, MgO-Av, and MgO-CP samples in the MIC test.

Some additional FTIR bands were found in the three synthesized oxides that resembled the FTIR bands found by Karthik et al. (2017) [4] at 3445, 1383, and 1638 nm. According to the authors, MgO was obtained with the same crystallographic phase by microwave-assisted green synthesis and calcining at 400 °C for 2 h. The results of the disk diffusion test for MgO obtained by Karthik et al. [4] were superior to the results reported in this work. As previously mentioned, the disk diffusion test is a qualitative method as it is limited by the mobility of the oxide in the disks and does not present the inhibition results as a concentration value. However, the disk diffusion test is a qualitative method as it is limited by the mobility of the oxide in the disk, and the inhibition results are not directly connected to the oxide concentration. Nevertheless, the MgO-St, MgO-CP, and MgO-Av samples in the MIC test showed higher inhibition against *E. coli*, *S. aureus*, and *C. albicans* than the samples reported in [45].

The sample morphology varied with the fuel used. MgO-St presented a sponge-like morphology, while MgO-CP and MgO-Av presented pseudo-spheres and lamellar morphologies, respectively. According to the literature [2,7], magnesium oxide presents several possible morphologies, depending on the synthesis method employed or the fuel used in the synthesis. In the work of Bindhu et al. [20], MgO nanoparticles were obtained via a chemical precipitation reaction using magnesium nitrate and sodium hydroxide calcinated at 400 °C for 5 h. The particles were almost spherical in shape, with smooth surfaces, and this morphology is very similar to that of MgO-CP. The antimicrobial activity studied by Bindhu et al. [20] was tested against *S. aureus* using the disk diffusion method, and the zones of inhibition were similar to those found in this work. However, the MgO nanoparticles synthesized by Bindhu et al. [20] were not tested against *E. coli*, but rather against the Gram-negative pathogen *Pseudomonas aeruginosa*.

The average particle diameter sizes for the synthesized samples ranged from 0.85 to 1.14 µm. For the MgO-St oxide, the average particle diameter size was 0.99 µm; this sample was the most porous, justifying its higher antimicrobial activity [36]. According to Ananda et al. [35], the presence of pores provides a large surface area, which supports high antimicrobial activity. In addition, this high activity can also be associated with the higher percentage of Mg ions in this sample, as a possible mechanism of antimicrobial action is the release of Mg^2+^ ions, as cited earlier.

Both the antimicrobial activity and the crystalline structures of our samples were very similar to those found in the literature; however, in this work, we used the lowest calcination time, only one hour, while other studies have reported calcination times greater than 5 h using traditional synthesis methods [20,44,45]. However, differences in the samples’ morphology can be observed, which may explain the differences in the antimicrobial activity, associated with the small particle size and high porosity, favoring a larger contact area.

## 4. Materials and Methods

### 4.1. Materials

Three different fuels were used to synthesize magnesium oxide: cassava starch (route I), citrus pectin (route II), and *Aloe vera* (route III). The cassava roots used for the extraction of starch were harvested in Palmital, Paraná, Brazil, and the *Aloe vera* leaves were harvested in São José, Paraná, Brazil. The other materials used were magnesium nitrate hexahydrate (Mg(NO_3_)_2_.6H_2_O, 98%, Dynamics) and commercial citrus pectin (Dynamics). The analytical reagents were of high purity, and all the solutions were prepared with deionized water.

The same calcination parameters were used in the three synthesis routes. First, the suspensions were calcined in a muffle furnace at a temperature of 750 °C with a heating ramp of 10 °C min^−1^ for 60 min. The products obtained were pulverized, sieved on a 250 mm (60 mesh) sieve, and stored in a suitable container for characterization and application. The MgO samples obtained by the three different synthetic routes were labeled MgO-St (Route I), MgO-CP (Route II), and MgO-Av (Route III).

### 4.2. Synthesis Using Cassava Starch (Route I)

To obtain MgO with the starch additive, the methodology used was adapted from Primo et al. [46]. Initially, 500 g of natural starch from manioc was extracted in 2500 mL of deionized water under mechanical agitation for 3 h. Then, the colloidal suspension was sieved and used as the base solution. The oxide MgO-St was synthesized from starch (300 g) and magnesium nitrate (64 g) and agitated for 20 min.

### 4.3. Synthesis Using Citrus Pectin (Route II)

Using citrus pectin as an organic and energetic precursor, MgO was synthesized using the gelation method, adapted from Dalpasquale et al. [12]. In the first step, 1500 mL of deionized water was heated to 80 °C, and 15 g of citrus pectin was added under constant agitation (800 rpm) until it was solubilized and a colloidal suspension formed. Magnesium nitrate salt (32 g) was added to the citrus pectin colloidal suspension, remaining under agitation and temperature control for 3 h.

### 4.4. Synthesis Using Aloe vera Barbadensis Miller (Route III)

To obtain the magnesium oxide prepared using the gel extracted from *Aloe vera* leaves, the synthetic route was adapted from Primo et al. [10]. First, the gel of the leaves was removed and processed in a Britania B1000 blender (power: 1200 W). The gel was sieved and then kept under refrigeration (2 °C). Thus, an *Aloe vera* gel broth extract with a concentration of 90% was prepared with deionized water, and the volume was 100 mL. Subsequently, magnesium nitrate (24 g) was dissolved in the aloe extract solution under constant magnetic stirring (60 min).

**Figure 6:**
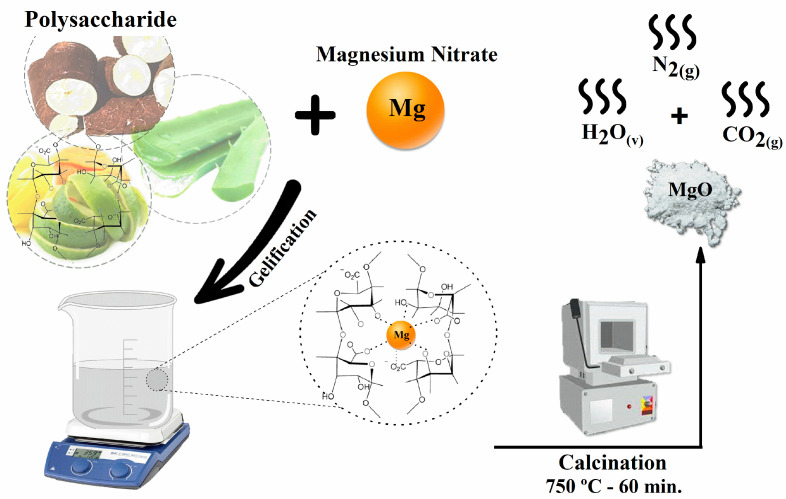
General schematic diagram of synthesis using polysaccharides as gelling complexing fuels.

Figure 6 represents the general scheme of synthesis using organic fuels for the synthesis of MgO. Metal ion complexation occurs by coordinating with the functional groups of the organic molecule, and, after calcination, there is not only the formation of the magnesium oxide but also the release of H_2_O vapor and N_2_ and CO_2_ gas [47].

### 4.5. Characterization Techniques

The elemental composition of the structured MgO was evaluated using an energy-dispersive X-ray spectrometer (EDX) (Shimadzu, Kyoto, Japan), model EDX-7000, containing an Rh tube, operating at 50 and 15 W. The crystalline structure and phase purity were characterized by X-ray diffractometry (XRD-D2 Phaser; Bruker, Billerica, MA, USA), with a copper cathode (λ = 1.5418 Å), 30 kV potential, 10 mA current, ranging between 10° and 80° (2θ) and with 0.2 °/s increments. We used Phase Identification from Powder Diffraction, Version 3.4.2, with access to the Crystallography Open Database (COD). Fourier transform infrared spectroscopy was performed in a Perkin Elmer Frontier device (Waltham, MA, USA). The samples were prepared in such a way as to obtain a pellet, which consisted of a mixture of a transparent matrix to which the sample was added; we used potassium bromide (KBr) that was previously dried and kept in an oven at 100 °C for 72 h until the time of maceration with the samples. We macerated 250 mg of each oxide to 25 mg of KBr until homogenization. The ground and homogenized mixture was deposited in a steel mold (Sigma-Aldrich Z506699-1EA, St. Louis, MI, USA) and subjected to pressure of approximately 3.0 Kg.cm^−2^ in a hydraulic press. With the procedure, 13-mm-diameter pellets were obtained for the formation of absorbance or transmittance spectra with a wider wavenumber. Samples were scanned from 450 to 4000 cm^−1^ at a spectral resolution of 4 cm^−1^. The morphology of the MgO particulate samples was examined with a scanning electron microscope (SEM-VEGA 3; TESCAN, Brun, Czech Republic); for the analysis, each sample was dispersed in water, and a drop of dispersion was deposited on an Al sample holder. The samples were gold-coated to render their surfaces conductive.

### 4.6. Antifungal and Antibacterial Tests

#### 4.6.1. Disk Agar Diffusion Method

The MgO samples were tested against *Candida Albicans* (ATCC 64548), *Escherichia coli* (ATCC 8739), and *Staphylococcus aureus* (ATCC 6538P) by disk diffusion using a methodology adapted from the Clinical and Laboratory Standard Institute [48]. The MgO sample disks were prepared with 250 mg of each solid oxide and deposited in a steel mold (Sigma-Aldrich Z506699-1EA) and subjected to pressure of approximately 3.0 Kg.cm^−2^ in a hydraulic press, forming disks of 13 mm diameter. The pathogens were cultured overnight and then diluted with saline solution (0.85%) to a concentration of 108 CFU.mL^-1^ (McFarland 0.5). The pathogen suspension was inoculated on the surface of Muller–Hinton Agar using a sterile swab. The sample disks of MgO-St, MgO-CP, and MgO-Av were placed on the agar surface and then incubated at 36 °C (±1) for 24 h. The tests were performed in triplicate, and the antibacterial and antifungal activity was evaluated by measuring the diameters of halos of growth inhibition strains assayed (in mm). The obtained data were analyzed by one-way analysis of variance (ANOVA) and *t*-test analysis. A value of *p* < 0.05 was considered to be statistically significant.

#### 4.6.2. Minimum Inhibitory Concentration (MIC) Assay

The antibacterial properties of the obtained oxides were also investigated by the minimum inhibitory concentration (MIC) test against *Staphylococcus aureus* (ATCC. 25923), *Escherichia coli* (ATCC 25922), and *Candida Albicans* (ATCC 64548). *E. coli*, *S. aureus*, and *C. albicans* inoculates were grown at 35 °C for 18 h and diluted to obtain a final well density of 105 CFU.mL^−1^. Solutions of different concentrations of each oxide were prepared individually and diluted in dimethyl sulfoxide (DMSO) to reach final concentrations ranging from 400 µg.mL^−1^ to 850 µg.mL^−1^, with a range of 50 µg.mL^−1^. In each well, 150 µL of Mueller–Hinton broth containing the inoculum and 50 µL of each dilution of MgO-St, MgO-CP, and MgO-Av oxides were added. The broth microdilution method was used according to the methodology adapted from the Clinical and Laboratory Standards Institute Manual [21] in 96-well microplates. The microplates were incubated at 35 °C for 24 h. Bacterial growth was confirmed by adding 10 μL of sterile aqueous solution (20 mg.mL^−1^) of triphenyl tetrazolium chloride (TTC, Inlab, Brazil) after incubation at 35 °C for 30 min. The bacteria reduced the TTC dye from yellow to red, indicating bacterial growth [49].

## 5. Conclusions

MgO samples were successfully obtained using three synthetic routes. The routes are efficient, reproducible, and low-cost methods that use suitable fuels from renewable sources. The single-phase periclase was identified for all MgO samples. The MgO-St sample showed crystallinity and the highest percentage of magnesium (61.7%). These results reflect the efficiency of controlling the microorganisms *Staphylococcus aureus* and *Candida albicans* seen in the agar disk diffusion method. Moreover, the MIC method showed the concentration of inhibition of the studied microorganisms. The most satisfactory results were observed for the antibacterial and antifungal tests with the MgO-St sample. This study presents three promising and low-cost synthesis methodologies for the preparation of antibacterial and antifungal MgO materials.

## Figures and Tables

**Table 1:** Elemental chemical compositions of the synthesized MgO samples and precursors used in the synthesis in element percent (% element) by EDXRF.

Sample	Elements (%)								
	Mg	Ca	Zn	Cu	K	Al	S	P	Others
Mg(NO_3_)_2_.6H_2_O	98.0	0.8	-	0.1	-	-	1.0	-	0.1
Starch	6.6	26.0	4.0	4.4	22.5	15.2	11.6	4.9	4.8
MgO-St	86.6	2.7	0.7	0.4	1.0	6.3	-	1.7	0.6
*Aloe vera*	0.1	20.9	1.0	3.4	0.5	-	1.2	-	72.9
MgO-Av	64.1	8.8	0.4	0.7	0.4	-	1.1	0.4	24.1
Citric pectin	1.7	21.8	2.2	2.1	52.6	7.7	4.9	1.4	5.6
MgO-CP	76.2	8.1	1.6	0.5	1.3	7.3	2.6	0.5	1.9

**Table 2:** Average mean zones of inhibition (in mm) produced by synthesized MgO samples on the test organisms.

Microorganism	Disk Diffusion Method		
	MgO-St	MgO-CP	MgO-Av
*Staphylococcus aureus*	2.86 ± 0.23 ^a^	2.43 ± 0.51 ^b^	2.20 ± 0.34 ^b^
*Escherichia coli*	0	0	0
*Candida albicans*	3.0 ± 0 ^a^	1.36 ± 0.11 ^b^	0.16 ± 0.28 ^c^

^a,b,c^ Results are represented as the mean ± standard deviation. Different lowercase letters in the same row indicate significant differences at *p* ≤ 0.05 by Tukey’s test.

## Data Availability

Not applicable.

## Supplementary Materials

The following supporting information can be downloaded at: https://www.mdpi.com/article/10.3390/molecules28010142/s1, Figure S1: Elemental analysis of the MgO samples MgO-ST, MgO-Av, and MgO-CP. The Identification was performed using the Mg kα line located at 1.25 keV; Figure S2: Elemental analysis of the organic precursors and magnesium salt. The percentage of Mg presented by the precursors was 6.6% for starch, 0.1% for *Aloe vera*, and 1.7% for citrus pectin. The Mg(NO_3_)_2_.6H_2_O salt used in the three synthetic routes showed purity of 98% of magnesium. Identification was performed using the Mg kα line located at 1.25 keV.

## Sample Availability

Samples of the compounds magnesium oxides (MgO-St, MgO-CP and MgO-Av) are available from the authors.
