# Supplementary material for: Polysaccharides as Green Fuels for the Synthesis of MgO: Characterization and Evaluation of Antimicrobial Activities
Source: Molecules. 2022 Dec 24;28(1):142. doi: 10.3390/molecules28010142 (PMC9822341; doi:10.3390/molecules28010142)
Supplement: Supplementary file 1 [file molecules-28-00142-s001.zip › molecules-2045939-SI.pdf]

SUPPLEMENTARY

Polysaccharides as green fuels for the synthesis of MgO: Characterization and evaluation of antimicrobial activities

Nayara Balaba[1], Silvia Jaerger[1], Dienifer F. L. Horsth [1,2], Julia de O. Primo[1], Jamille de S. Correa[1], Carla Bittencourt[2*], Cristina M. Zanette[3], Fauze J. Anaissi[1]

[1] Departamento de Química, Universidade Estadual Do Centro-Oeste, 85040-080 Guarapuava, Brazil

[2] Chimie des Interactions Plasma-Surface (ChIPS), Research Institute for Materials Science and Engineering, University of Mons, 7000 Mons, Belgium

[3] Departamento de Engenharia de Alimentos, Universidade Estadual do Centro-Oeste, 85040-080 Guarapuava, Brazil

*E-mail: carla.bittencourt@umons.ac.be

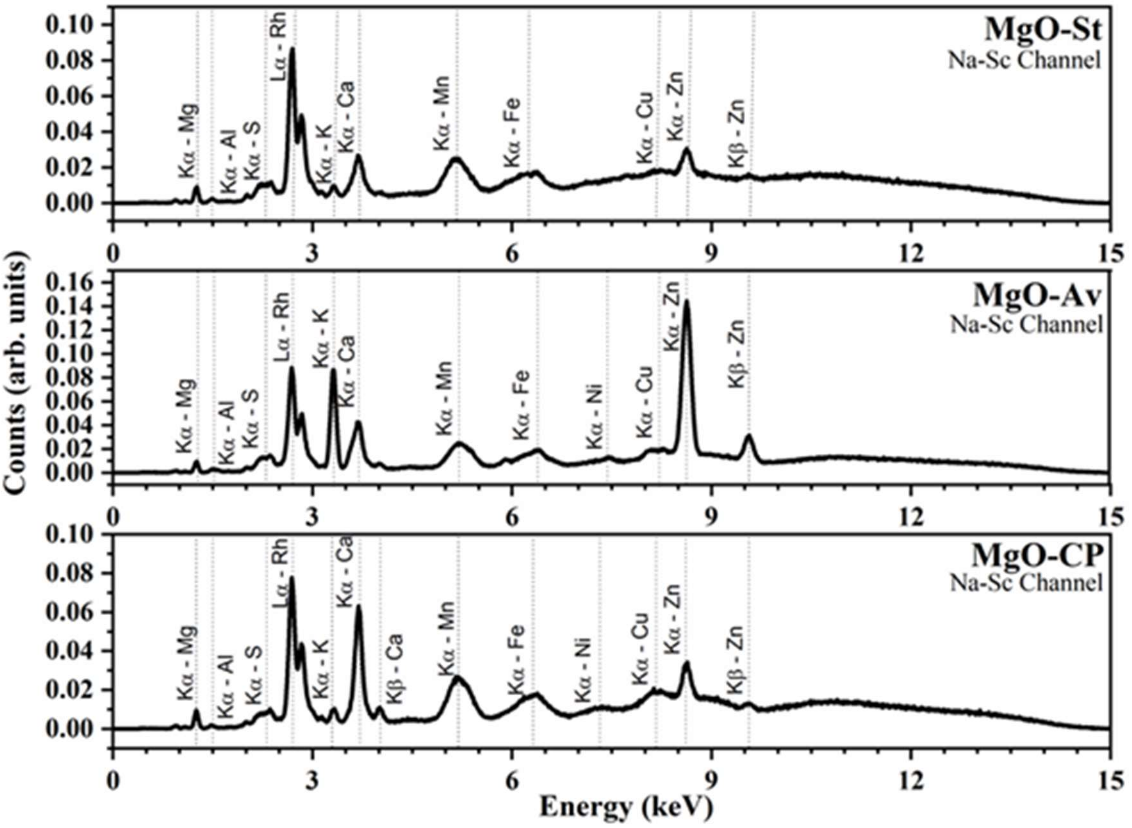

Figure S1. Elemental analysis of the MgO samples MgO-ST, MgO-Av, and MgO-CP. The Identification was performed using the Mg kα line located at 1.25 keV.

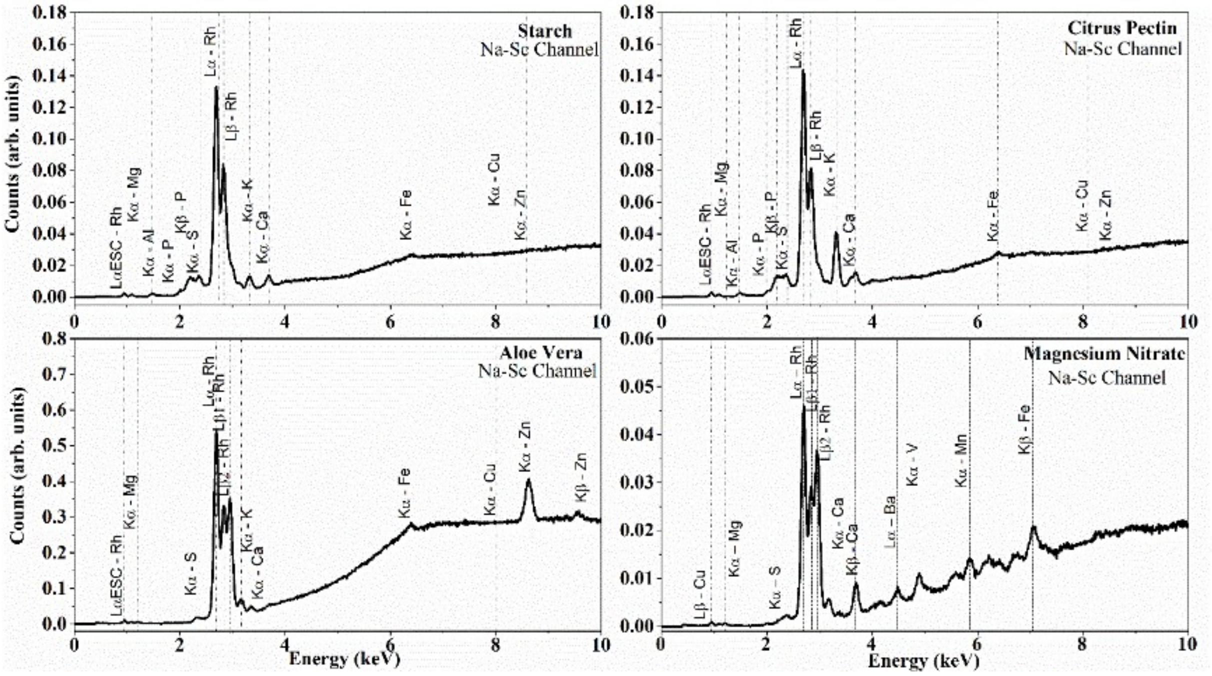

Figure S2. Elemental analysis of the organic precursors and magnesium salt. The percentage of Mg presented by the precursors was: 6.6% for starch, 0.1% for Aloe Vera, and 1.7% for citrus pectin. The $Mg(NO_3)_2.6H_2O$ salt used in the three synthetic routes showed a purity of 98% of magnesium. Identification was performed using the Mg kα line located at 1.25 keV.
